# Estimating Health-Related Quality of Life Based on Demographic Characteristics, Questionnaires, Gait Ability, and Physical Fitness in Korean Elderly Adults

**DOI:** 10.3390/ijerph182211816

**Published:** 2021-11-11

**Authors:** Myeounggon Lee, Yoonjae Noh, Changhong Youm, Sangjin Kim, Hwayoung Park, Byungjoo Noh, Bohyun Kim, Hyejin Choi, Hyemin Yoon

**Affiliations:** 1 Center for Neuromotor and Biomechanics Research, Department of Health and Human Performance, University of Houston, Houston, TX 77004, USA; mlee47@central.uh.edu; 2 Department of Management Information Systems, Dong-A University, Busan 49236, Korea; kar98n@donga.ac.kr (Y.N.); yoonlea1205@donga.ac.kr (H.Y.); 3 Department of Health Sciences, The Graduate School of Dong-A University, Busan 49315, Korea; app00113@donga.ac.kr (H.P.); 2177638@donga.ac.kr (B.K.); chjin0907@donga.ac.kr (H.C.); 4 Department of Kinesiology, Jeju National University, Jeju 63243, Korea; bnoh@jejunu.ac.kr

**Keywords:** health-related quality of life, physical and mental components, elderly adults, machine learning, prediction model

## Abstract

The elderly population in South Korea accounted for 15.5% of the total population in 2019. Thus, it is important to study the various elements governing the process of healthy aging. Therefore, this study investigated multiple prediction models to determine the health-related quality of life (HRQoL) in elderly adults based on the demographics, questionnaires, gait ability, and physical fitness. We performed eight physical fitness tests on 775 participants wearing shoe-type inertial measurement units and completing walking tasks at slower, preferred, and faster speeds. The HRQoL for physical and mental components was evaluated using a 36-item, short-form health survey. The prediction models based on multiple linear regression with feature importance were analyzed considering the best physical and mental components. We used 11 variables and 5 variables to form the best subset of features underlying the physical and mental components, respectively. We laid particular emphasis on evaluating the functional endurance, muscle strength, stress level, and falling risk. Furthermore, stress, insomnia severity, number of diseases, lower body strength, and fear of falling were taken into consideration in addition to mental-health-related variables. Thus, the study findings provide reliable and objective results to improve the understanding of HRQoL in elderly adults.

## 1. Introduction

Improved living conditions and developments in medicine and technology have increased longevity globally [1]. The proportion of the elderly population in South Korea has increased substantially, accounting for 15.5% of the total population in 2019 [2]. Over the past century, the gradual increase in the elderly population worldwide has increased the interest of researchers in the concept of aging well. According to the World Health Organization, healthy aging is defined as “the process of developing and maintaining the functional ability that enables well-being in older age”. This process spans the entire life course and is considered relevant to every individual, including patients and those free from diseases [3]. Additionally, the primary concept of aging well involves minimizing the deterioration of the physical and mental health [4], thus maintaining functional ability and well-being with aging [3,5].

Quality of life (QoL) is a multidimensional concept, which can be assessed based on the physical and mental health, independence, social relationships, beliefs, and relationship with salient features of the environment [6]. Particularly, independence in daily life and several diseases are significant factors affecting the QoL in elderly adults [5,6,7,8]. The 36-item, short-form health survey (SF-36) is a commonly used questionnaire with 36 questions that can evaluate the health-related quality of life (HRQoL) in terms of both physical and mental components [5,9,10]. Moreover, the decline in the HRQoL in elderly adults may affect younger populations as a social and economic burden. Therefore, it is necessary to understand the factors governing HRQoL and strategize the improvement and maintenance of physical and mental health in elderly adults.

The HRQoL can be aggravated by diseases [6,7], fallings [11,12,13], physical activity (PA) levels [14,15], muscle strength [16,17,18], and gait ability [5] in elderly adults. For instance, a slower walking speed in elderly adults reflects decreased muscle strength, which may increase the falling risk [19,20]. Additionally, low handgrip strength reflects low QoL in elderly adults [16,17,18]. Therefore, slower walking speed and lower handgrip affect the risk of mortality in elderly adults [17,21]. To improve the HRQoL in the elderly population, several researchers recommended enhancing the PA levels and conducting exercise interventions [22,23].

In summary, existing studies have reported the relationship between the status of HRQoL and potential factors that influence the QoL in elderly adults. However, most studies relied on simple variable approaches, such as demographics vs. HRQoL, questionnaire vs. HRQoL, gait ability vs. HRQoL, and physical fitness vs. HRQoL, rather than a multidisciplinary approach. This limitation induces the need to deduce the potential factors that are more important or essential variables to evaluate the status of HRQoL in elderly adults. Machine learning (ML) figures out patterns through data and makes predictions based on enormous computation, and predictive models based on the ML algorithms devised by outstanding domain experts and data scientists may discover new meanings and insights hidden in data [24]. Based on these advantages, several studies recently predicted HRQoL in patients and the elderly using ML methods to detect influencing factors for the HRQoL [5,25,26]. However, only one study used a multidisciplinary approach to predict HRQoL in adults aged over 45 years, reporting that handgrip strength is a powerful indicator of HRQoL in elderly adults [5]. This study considered multiple variables, such as demographic, medical, and physical performances, which generated meaningful results to understand the HRQoL. However, as the range of age in the variance of samples was composed of middle-aged adults of over 45 years, it was not suitable for prediction models of HRQoL in elderly adults. Additionally, they did not consider essential tasks to evaluate functional capacities. Another study assessed four physical fitness domains, such as strength, flexibility, balance, and endurance, to evaluate elderly adults [27]. Furthermore, gait tasks were conducted considering challenging conditions, such as slower or faster than self-preferred walking speeds, to provide advanced insight into the understanding of dynamic stability. Moreover, several studies reported significant results based on individual gait tasks under slower or faster walking speed conditions [27,28,29,30,31]. This implies that prediction models that evaluate HRQoL in elderly adults based on demographic characteristics, questionnaires, gait ability, and physical fitness can be a potential reference in clinical environments.

Therefore, we investigated multiple prediction models in this study to identify the optimal model for determining the HRQoL, including both physical and mental health components, in elderly adults. We considered the demographic characteristics, questionnaires, gait ability, and physical fitness using the ML approach and determined the factors that can effectively evaluate the HRQoL in elderly adults.

## 2. Materials and Methods

### 2.1. Study Participants

We recruited 891 elderly adults aged between 65 and 89 years as participants of a community-wide survey in Busan from February to December 2018. However, 109 participants were excluded from the study owing to personal reasons (n = 29), non-completion of the 1-min treadmill walking test at three different speeds (n = 23) and physical fitness tests (n = 33), and withdrawal of informed consents (n = 24). In total, 782 elderly adults comprising of 252 and 530 men and women, respectively, participated in the study. However, seven samples were eliminated during the preprocessing stage because of a missing value in the “single-leg stance” test. Therefore, a total of 775 participants were considered in the final study (male = 251; female = 524). We ensured that the participants had no history of musculoskeletal or neurological problems that affected the gait and that they were capable of walking without any support during the previous six months. The relevant guidelines and regulations were followed during the execution of all methods, and all participants signed their informed consents after reading the study details. This study of Dong-A University was approved by the Institutional Review Board (IRB number: 2–104709–AB–N–01–201808–HR–023–02).

### 2.2. Instrumentation

Shoe-type inertial measurement unit (IMU) systems (DynaStab™, JEIOS, Busan, Republic of Korea) with shoe-type data loggers (Smart Balance SB-1^®^, JEIOS, Busan, Republic of Korea) and a data-acquisition system were used in this study. The shoe-type data logger included an IMU sensor (IMU-3000™, InvenSense, San Jose, CA, USA) on the outsoles of both shoes to measure the triaxial acceleration and angular velocities along the three orthogonal axes. The data were transmitted wirelessly to a data-acquisition system via Bluetooth^®^ [32,33,34]. Additionally, the shoe sizes ranging from 225 to 280 mm were adapted to fit the study participants.

### 2.3. Test Procedure

All test procedures, such as the measurement of the demographic characteristics, questionnaire surveys, SF-36 tests, physical fitness tests, and gait tasks, were completed in a single day.

#### 2.3.1. Evaluating QoL Using SF-36

SF-36 is commonly used to measure HRQoL in terms of physical and mental health constructs [9,10]. The SF-36 questions can measure eight subscales:

1. Physical functioning;
2. Role limitations owing to physical health;
3. Role limitations caused by emotional problems;
4. Pain;
5. General health;
6. Vitality;
7. Emotional well-being;
8. Social functioning.

The eight scales aggregated two HRQoL components, namely the physical and mental health components [9,35], which were scored from 0 to 100 [10]. A high score reflects a better QoL, whereas a low score reflects a poor or suboptimal QoL [8].

#### 2.3.2. Demographic Characteristics

Demographic characteristics include sex, age, body height, weight, body mass index (BMI), waist and hip circumstances, waist-to-hip ratio, and physical composition, such as body fat percentage, muscle and body fat mass (InBody 270, InBody Co. Ltd., Seoul, Republic of Korea), and blood pressure. Additionally, we considered the participants’ education level, job, residential environment, household members, smoking and drinking habits, and social relationships. Furthermore, the total number of diseases and medication doses were recorded. The diseases included cerebrovascular diseases, hypertension, hyperlipidemia, diabetes, gastrointestinal disorder, cardiovascular diseases, liver diseases, respiratory diseases, glaucoma or cataract, osteoporosis, low back pain, and knee and hip joints pain; medication doses included sleeping, painkiller, and antipsychotic pills. Therefore, the total number of diseases and use of medical drugs were considered for the analysis with yes = 1 and no = 0.

#### 2.3.3. Assessment Using Other Questionnaires

All the participants answered questionnaires to assess their PA levels, insomnia severity index (ISI), and stress response. The PA level was evaluated using the international PA questionnaire short form, and the metabolic equivalents (METs/week) were calculated [36]. The severity of insomnia was evaluated using an ISI questionnaire, comprising of seven questions assessing the severity of sleep onset, sleep maintenance difficulties, and satisfaction with the current sleep [37]. The collected answers were added to obtain the total score, which was used to determine the severity of insomnia [37,38]. Additionally, a mini-mental state examination questionnaire was used to assess the global cognitive function [39]. Finally, the stress response was assessed using the modified stress response inventory (SRI-MF), which involved 22 questions; a higher score of SRI-MF indicates severe stress levels [40]. Furthermore, all participants answered questions pertaining to fall history, such as whether they have fallen in the last six months, number of falls, and fear of falling.

#### 2.3.4. Physical Fitness Test

We assessed four domains underlying physical fitness, namely strength (upper/lower body), flexibility (lower body), balance (static/dynamic), and functional or cardiorespiratory endurance. All the participants completed eight physical fitness tests in the following order (Table S1):

1. Grip strengths of both the hands were measured using an isometric digital handgrip dynamometer (T.K.K. 5401 Grip-D, Takei Scientific Instruments, Tokyo, Japan) to assess the upper body strength;
2. Upper body strengths of male and female participants were assessed based on the bicep curls performed using dumbbells weighing 3 kg and 2 kg, respectively;
3. Lower body strength was assessed considering the performance of five times sit-to-stand exercises;
4. Standing time (ST) from a long sitting position (LSP) was measured to assess the lower body strength;
5. Chair sit-and-reach test was conducted to assess the lower body flexibility;
6. Single-leg balance (dominant leg) was conducted to assess the static balance;
7. A 3-m timed-up-and-go test was conducted to assess the dynamic balance;
8. A 6-min walk test (6MWT) was conducted to assess the functional or cardiorespiratory endurance.

**Figure 1:**
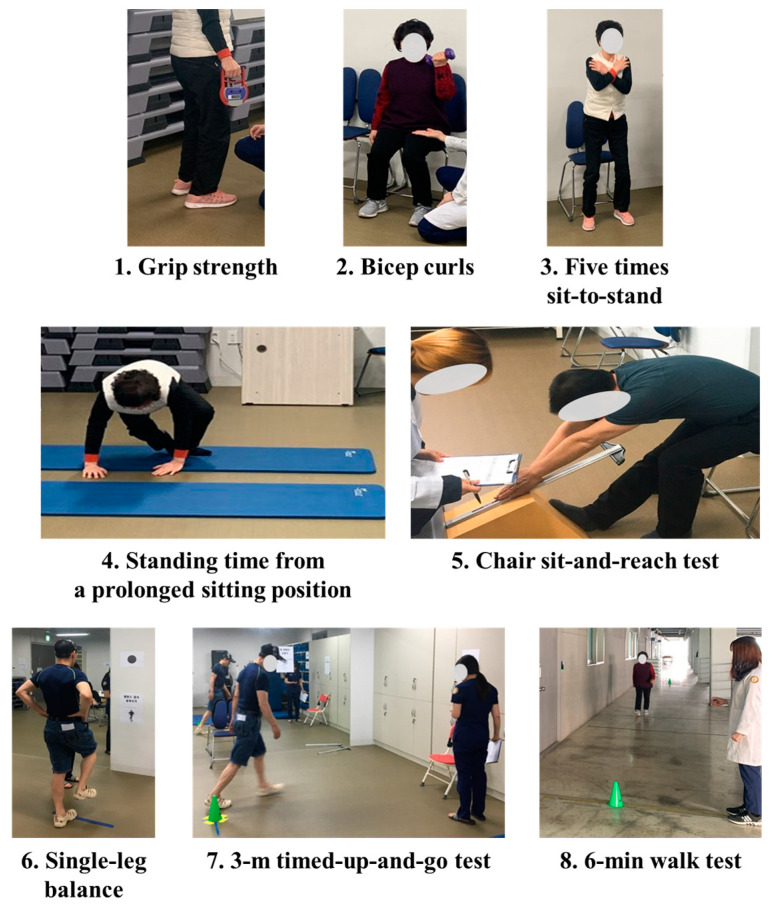
Physical fitness test.

The mean scores were calculated for two attempts of each physical fitness test, as depicted in Figure 1 (see Table S3) [27].

#### 2.3.5. Overground Walking Test at Different Speeds

The overground walking test along a straight 20-m walkway at 80% of preferred (slower), self-preferred (preferred), and 120% of preferred (faster) walking speeds were performed three times based on previous studies (see Table S3) [27,28]. The preferred speed can be defined as the normal walking speed when performing daily activities without any support during overground walking. The slower and faster walking speeds were calculated relative to the preferred speed, which was quantified using a metronome (beats/min) [27,28]. Participants were instructed to perform the overground walking test at speeds as close as possible to the target walking speeds. Although natural stepping rhythms and rhythmic metronome beats did not concur perfectly, we attempted to control the accuracy of each participant’s performance to the highest extent [27]. The participants practiced all speed conditions before the actual tests by walking once or twice with the metronome, and the metronome was switched off during the test trials.

### 2.4. Data Analysis

The overground walking data were filtered using a second-order Butterworth low-pass filter with a cut-off frequency of 10 Hz [32,33,34]. A heel strike can be defined as an event where the linear acceleration on the anteroposterior axis attains its maximum positive value, whereas a toe-off event occurs when the linear acceleration on the vertical axis attains its maximum positive value during the gait cycle [33,34].

We calculated the spatiotemporal parameters, such as the walking speed, stride length, step length, single-support phase, double support phase, stance phase, cadence, stride time, and step time [41]. Additionally, the values of the percentage coefficient of variance (CV) ((standard deviation/mean) × 100) were calculated to determine the gait variability (GV) for all the spatiotemporal parameters.

### 2.5. Statistical Analysis

**Figure 2:**
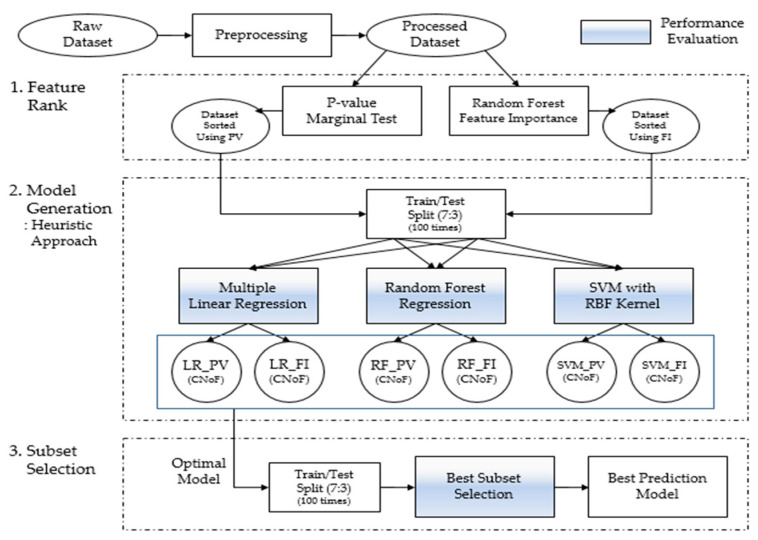
Scheme of the study.

Figure 2 depicts the scheme of this study, comprising three layers of procedures:

1. To identify the dominance of features, we used the feature selection ranking method, including the *p*-value (PV) [42] and feature importance (FI) based on the random forest (RF) method [43];
2. Models are generated using multiple linear regression (LR), RF [44], and support vector machine (SVM) with a radial basis function kernel [45]. The optimal model was selected via heuristic learning based on the feature selection ranking methods;
3. Subset selection [46] was achieved using the number of features in the optimal model to determine the best combination of features that maximizes the performance.

Root mean square error is one of the criteria used to evaluate supervised learning in terms of predictions.

To determine the robustness of performance, each model was trained and evaluated based on 100 resampling procedures, each of which divided the sampled datasets into training and testing sets of 70% and 30%, respectively. The performance of the model was evaluated using the average root mean square error (aRMSE), derived from the results of repeating the procedure 100 times; aRMSE can be calculated as follows:

(1) $${\mathrm{aRMSE}}_{model}=\frac{1}{100}\sum_{j=1}^{100}\sqrt{\frac{1}{n}\sum_{i=1}^{n}{\left\{y_{i,j}^{test}-f_{model}\left(X_{j},Z_{j}\right)\right\}}^{2}},$$

where *i* denotes the test sample number, *j* indicates the seed number, and $f_{model}\left(X,Z\right)$ represents the estimator of test data X using a model trained by the training dataset Z.

Additionally, to ascertain the characteristics of the features of the best subset, we analyzed the subsets using a Pearson correlation matrix and network analysis [47].

#### 2.5.1. Data Setup

Raw datasets comprised of 782 participants with 98 variables. As mentioned in Section 2.1, 7 samples were excluded because of missing values. Furthermore, 2 features of systolic blood pressure and diastolic blood pressure were excluded. Therefore, the preprocessed data matrix comprised of 775 samples and 96 features, each of which was standardized using Gaussian distribution.

#### 2.5.2. Feature Rank

We used two filtering methods, namely PV and FI, in this study [42,43,48].

1. To rank high-dimensional features, feature-by-feature statistical testing with analysis of variance and simple LR for categorical and continuous data, respectively, was performed. The PV-based marginal test, which is a type of filter method, is a pairwise calculation that focuses on the main effect of the feature on the target individually and not the joint effect of the features [48]. After calculating the PVs of the features, we sorted the list in the ascending order (Table S2).
2. The FI in RF, computed based on the Gini importance, was used as another feature ranking method [43]. It is a representative ensemble-based ML methodology, with the concept originating from predictive model construction by combining trees [49]. FI considers both the interaction effect among features and the main effect of the feature on the target individually [49,50]. After clarifying the FI, it was arranged in the descending order of the FI (Table S2).

#### 2.5.3. Model Generation: Heuristic Approach

We deployed three ML models, namely LR, RF, and SVM. Each model was generated using the feature ranking methods based on both PV and FI by accumulating the features individually. After the performance evaluation, we developed the model with a cumulative number of features (CNoF) minimizing the aRMSE (see File S1).

#### 2.5.4. Subset Selection

To identify the best prediction model, the models were fitted to all possible combinations of the features in the model, minimizing the aRMSE. It can reduce the error of prediction by sacrificing some features [46]. After reviewing the models, the best prediction model that clarifies the feature combination minimizing the aRMSE was identified.

## 3. Results

### 3.1. Model Comparison

**Figure 3:**
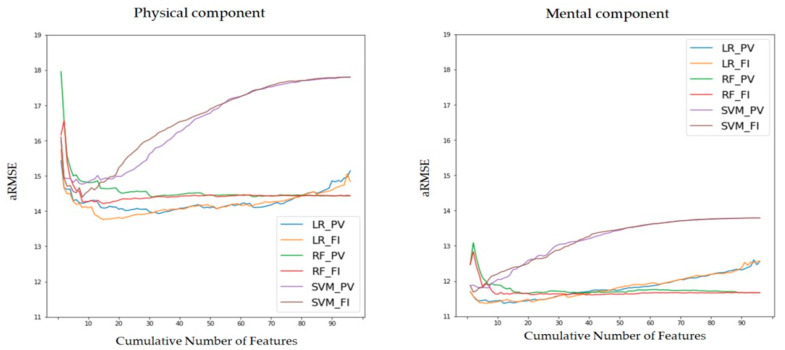
Average root mean square error (aRMSE) based on the cumulative number of features (CNoF) of the six categorized models. The y-axis indicates the aRMSE of the model; the x-axis indicates the level of the CNoF sorted by the ranking of feature selection methods. Each line graph indicates one of the six categorized models that is identical to the legend. LR_PV, multiple linear regression (LR) model with feature rank based on *p*-value (PV); LR_FI, multiple LR model with feature rank based on feature importance (FI); RF_PV, random forest (RF) regression with feature rank based on PV; RF_FI, RF regression with feature rank based on FI; SVM_PV, support vector machine (SVM) regression of radial basis function kernel with feature rank based on PV; SVM_PV, SVM regression of radial basis function kernel with feature rank based on FI.

Using the model building method, a total of 2 × 3 × 96 number of models was generated. Each of aRMSE from the model was calculated using Equation (1). The feature ranking methods and ML models used in the study can be categorized into the six combinations. Figure 3 illustrates the aRMSE levels according to CNoF with respect to various models. Although there are some differences in performance depending on the feature ranking method, an aRMSE change shows a similar pattern based on the ML methods.

As depicted in Figure 3, the aRMSE tends to fluctuate in the case of the LR model, initially decreasing and then increasing with an increase in the CNoF. Conversely, in the case of the RF model, the aRMSE begins at a high level and decreases drastically at approximately 10 CNoF; as the CNoF increases, the aRMSE tends to converge. In the case of SVM, which exhibits the lowest performance, the aRMSE is minimized under 10 CNoF and increases steadily as the CNoF increases.

**Figure 4:**
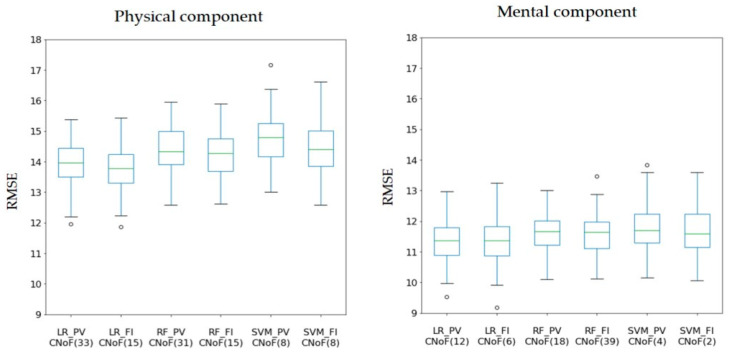
Box plots of root mean square error (RMSE) with respect to the model with its CNoF minimizing the aRMSE. It depicts the boxplots of RMSE distribution in terms of the CNoF that minimizes the aRMSE. CNoF, cumulative number of features, wherein the number in the parentheses indicates the CNoF maximizing performance within each category (Table 1); green lines in the middle of the boxplot indicate the median.

Table 1 summarizes the optimal CNoF level, aRMSE, standard deviations in the RMSE (std RMSE), and the quartile of RMSE of different models (Table S2). Figure 4 illustrates the distribution of RMSE with box plots, which comprises of aRMSE under the CNoF minimizing the aRMSE.

Based on these outputs, the best performing model satisfies the minimum aRMSE value among the different values computed by all the possible models.

1. In the case of the physical component, we concluded that the LR model with the FI feature rank is the best performing model with a CNoF of 15. Additionally, this model exhibits dominant statistics, i.e., the aRMSE and median. Conversely, the worst model among the six models in Table 1 is the SVM with the PV feature rank, wherein the CNoF is 8.
2. In the case of the mental component, we figured that the LR model with the PV feature rank is the optimal model with a minimum aRMSE and with a CNoF of 12. However, the worst model among the six models in Table 1 is the SVM with the PV feature rank and its CNoF is 4.

### 3.2. Best Prediction Model

We determined the best performing model and its respective CNoF via model generation. The total number of subsets of its physical and mental components are $2^{15}$ − 1 and $2^{12}$ − 1, respectively. The minimum aRMSE was calculated through simulations of the best subset selection, which decreased the level of the aRMSE for the best performing model in Section 3.1 from 13.76088 to 13.67993 (std RMSE = 0.71684) for the physical component and from 11.36805 to 11.27420 (std RMSE = 0.63343) for the mental component (Table S2).

### 3.3. Input Features of the Best Prediction Model

Table 2 summarizes the beta of features comprising the CNoF of the best performing model in Section 3.1. The selected features of the best prediction model are indicated with * in Table 2; the features are arranged in the order of the feature rank methods according to their physical and mental components, respectively.

**Figure 5:**
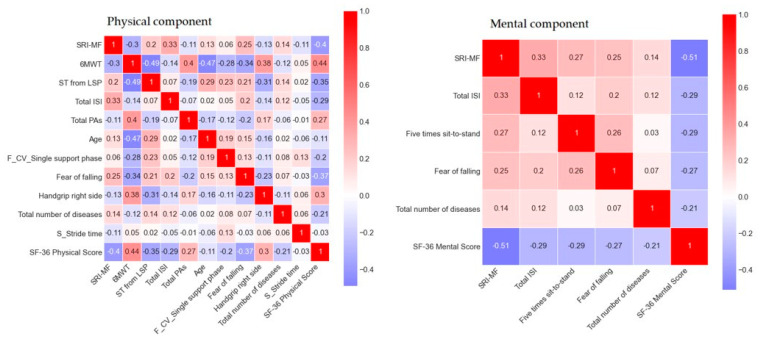
Heat-map of the variables in the best prediction model obtained from the Pearson correlation coefficient matrix, which is illustrated by color based on the correlation level. When the correlation levels are close to +1 and −1, the colors of the boxes are close to red and blue, respectively.

**Figure 6:**
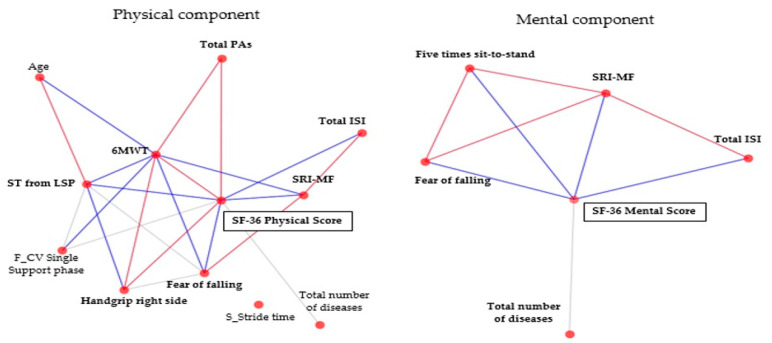
Network analysis of the edges based on the Pearson correlation. Edges in black, red, and blue represent |0.2| < r < |0.25|, r ≥ 0.25, and r ≤ −0.25, respectively.

To identify the characteristics of the features of the optimal model, we examined the relationship between the features and target. The correlation heat-map of the target and features (Figure 5) and network analysis (Figure 6) were obtained by calculating the Pearson correlation. Based on the heat-map in the case of the physical component, the “6MWT” and the target exhibited the highest correlation of 0.44. Furthermore, this feature highly correlated with “SRI-MF” (r = −0.40), “Fear of falling” (r = −0.37), “ST from LSP” (r = −0.35), and “Handgrip right side” (r = 0.30). Conversely, in the mental component, the “SRI-MF” and mental score exhibit the highest correlation of −0.51.

The network analysis, performed by modeling variables as nodes and the relationship between variables as the edges, determines the inter-relationships among objects. In this study, the edges are considered as Pearson correlation coefficients. In terms of the Pearson correlation, the SF-36 physical score is significantly affected by seven features underlying the physical component, which include “6MWT”, “Total PAs”, and “Handgrip right side” (with positive effect), “Total ISI”, “SRI-MF”, “Fear of falling”, and “ST from LSP” (with negative effect). Conversely, in the case of the mental component, the SF-36 mental score is primarily influenced by four features with a negative effect, including “Total ISI”, “SRI-MF”, “Fear of falling”, and “Five times sit-to-stand” (Figure 6).

## 4. Discussion

We analyzed the 775 samples of elderly adults using the ML algorithms based on the 96 independent variables, such as the demographic characteristics, questionnaires, gait ability, and physical fitness, to identify the optimal features that can effectively predict the QoL in terms of the physical and mental components. During the processing, we figured that the LR model showed the best performance in the significant interval in using the feature selection technique. The LR with the FI was selected as the best model for the physical component with a CNoF of 15; and the LR with the PV was selected as the best model for the mental component with a CNoF of 12; both models indicated the lowest aRMSE values. Additionally, the input features of each best model, which was derived by applying the best subset method, were indicated by the 11 best variables out of 15 in the physical component; additionally, the best model was indicated by the five best variables of 12 in the mental component. The primary findings of this study can be summarized as follows:

1. In terms of the physical component, demographic characteristics (age and the total number of diseases), questionnaires (total PAs, total ISI, and total SRI-MF and fear of falling), gait ability (CV of single-support phase at faster speed and stride time at slower speed), and physical fitness (walking distance during 6MWT, ST from LSP, and handgrip) features were identified as significant variables.
2. In terms of the mental component, demographic characteristics (total number of diseases), questionnaires (total ISI, total SRI-MF, and fear of falling), and physical fitness (five times sit-to-stand) features were identified as significant variables.
3. In particular, the 6MWD, ST from LSP, SRI-MF, fear of falling, and handgrip (r = 0.30–0.44) were highly correlated with the physical component; the total SRI-MF (r = −0.51) was the most highly correlated feature with the mental component. These findings are discussed in detail in the subsequent sections.

### 4.1. Feature Selection and Machine-Learning Model

The performance of an algorithm is dependent on significant feature selection relevant to target of interest. [51]. As the framework of our study demonstrates, we designed the ML with feature selection to improve both performance and explainability. Feature selection is an important procedure to obtain statistically significant factors for the power of performance [52,53]. Recently, several works on QoL in other fields have also focused on the feature selection while not only concentrating on the prediction power of the model but also detecting the important features affecting the target feature [54,55]. In this study, PV method focuses on the main effect of the feature on target marginally [48], while FI based on RF focuses on joint effect among features [49,50]. In terms of ML methodologies, LR model considers the relationship between independent variables and the target variable as a linear combination [56]. The main disadvantage of a single decision tree is overfitting of the training data, and the RF method prevents such overfitting [57]. As shown in Figure 3, these characteristics were also well shown in the framework of this study. We derived the best performing model by combining the two different cases of feature selection and the ML methodologies, such as LR, RF, and SVM.

Furthermore, the subset selection was also considered to select most significant features as well as the optimal model leading to the minimum aRMSE considering all the possible subset of features included in the selected best model [46]. As aforementioned, the aRMSE based on the result of the 100-times resampled data was utilized as a metric to determine the best model. It is guaranteed for the selected best model to be robustness. Therefore, it turns out that our ML models based on the LR with the FI and the LR with the PV can suitably predict the physical and mental components of the HRQoL in elderly adults, respectively.

### 4.2. HRQoL in Elderly Adults Based on the Physical Component

The results obtained from the ML techniques verified that demographic characteristics, questionnaires, gait ability, and physical fitness are the significant variables in terms of the physical component in elderly adults. Particularly, 11 variables were used to form the best subset of the features in the physical component. The demographic and questionnaire characteristic features, such as the SRI-MF score, total ISI score, fear of falling, total number of diseases, and age, were considered significant. The SRI-MF is a questionnaire that evaluates stress levels; it comprises several subscales, such as somatization, anger, and depression. The higher SRI-MF total score indicates severe stress levels [40]. Additionally, the ISI is a questionnaire that assesses the severity of sleep onset, sleep maintenance difficulties, and satisfaction with current sleep [37]. The higher ISI total score reflects severe insomnia status [37,38]. Previously, studies have reported that poor mental health and quality of sleep or severity of insomnia are associated with lower physical functionality in elderly adults [28,58]. Furthermore, several studies reported that the QoL can be associated with chronic diseases [6,8]. With an increase in the number of diseases, the QoL in elderly adults decreases [7]. Particularly, individuals with chronic non-communicable diseases, such as diabetes, high blood pressure, obesity, cancer, asthma, osteoarthritis, smoking, and excessive alcohol consumption, indicated lower scores of physical and mental components [6,8]. Additionally, fear of falling is one of the most common psychological concerns, and it can lead to modified behavior as well as restriction of activity in elderly adults [59,60,61]; several studies reported higher fear of falling may be associated with a lower HRQoL for the physical component [11,13]. Therefore, we conclude that the physical component of the HRQoL in elderly adults can be associated with the severity levels of stress, insomnia, diseases, and fear of falling, which concurs with the results of existing studies.

Poor physical functions, such as slower walking speeds and weaker muscle strength, are associated with a lower HRQoL for physical and mental status in elderly adults [18]. Typically, variables such as walking speed, handgrip, and 6MWD are used to evaluate the physical functions in elderly adults [18]. For instance, slower walking speed reflects weaker muscle strength and increased risk of falling in elderly adults [19,20]. Moreover, 6MWD is a simple and reliable test, and the maximum distance covered during this test can be used to evaluate the functional exercise capacity [62,63,64]. Additionally, the 6MWD is a useful task, as it is similar to daily activities [63]. Several studies have used this task on both pathological patients [62] and healthy elderly adults [63,64,65]. The performance during the 6MWD can be affected by age, sex, body weight and height, obesity, muscle strength, and disease factors [62,63,64,65,66]; relatively lower 6MWD value in the patients may reflect the risk of mortality [62,63]. Consequently, the walking speed is a predictor of mortality in elderly adults [21]. Furthermore, PA levels and exercise interventions can reduce the risk of falling, improving the HRQoL in terms of both the physical and mental components [23].

A slower walking speed affects the lower gait quality in elderly adults with increased falling risks [67], which may reduce the HRQoL. Recently, several studies suggested utilizing advanced gait-related variables, such as the GV and coefficient values for the spatiotemporal parameters, to evaluate the dynamic stability in elderly adults [68,69]. Particularly, gait phases are essential variables to evaluate the gait stability during one gait cycle, which is the duration from one stride to the subsequent stride. A slower walking speed and shortened stride length can lead to longer double support and stance phases and a shorter single-support phase [70], which indicate the weakness of the lower limbs [19,20]. The GVs for the gait phase are particularly useful variables, and the increased GV values may reflect a relatively low dynamic stability during walking [19,68]. In this study, the GV for the single-support phase at a faster speed and stride time at a slower speed were a significant independent variable for the physical component in elderly adults. A challenging task, such as faster or slower than self-preferred walking speed conditions, requires more mechanical energy with increased muscle activations [71]. It also requires an increased cognitive load during walking tasks, which reduces the gait automaticity in elderly adults [69]. The reduced gait automaticity can be associated with an increased GV value based on the stride-to-stride fluctuations, which indicates a decreased dynamic stability [72]. Similar investigations conducted on challenging walking tasks reported that the GV for the gait phase is a significant variable to evaluate the gait ability in patients with Parkinson’s disease [28] or healthy individuals. It can indicate reduced functions, such as cognitive functioning [30], subthreshold insomnia severity [29], and functional movement ability [31]. Particularly, the changes in GV can be associated with the fear of falling in elderly adults [19,73], and the GV value indicates a high falling risk when elderly adults walk at slower speed [41]. Thus, the GV value reflects gait ability and contributes to a negative effect for maintaining independence in elderly adults, which may reduce their QoL. Therefore, the GV for the gait phase during the faster speed condition can serve as a useful variable for evaluating the physical component of the HRQoL in elderly adults.

Furthermore, the handgrip strength is a valid variable to evaluate the general health status. It serves as an indicator in both healthy elderly adults and patients. A low handgrip strength can be associated with the QoL of elderly adults, which may affect an early all-cause mortality, cardiovascular mortality, disability, and sarcopenia [16,17,18]. A previous study suggested that individuals with low handgrip strength should undergo exercise interventions, such as resistance training, to improve their QoL [16]. Our study demonstrated that handgrip strength is an important independent variable to evaluate the physical component of the HRQoL in elderly adults, which concurs with the results of previous studies [16,17,18]. We determined that the ST from LSP is a significant variable for evaluating the physical component of HRQoL in elderly adults. Furthermore, as sitting on the floor is a life characteristic in East Asian countries, such as Korea and Japan [74], standing from a sitting position on the floor may be a familiar motion. Therefore, this is a more appropriate task than the sit-to-stand from the chair task to assess the lower body strength in Korean elderly adults.

Our results demonstrated that the top 10 features indicated significant independent variables to predict the status of the physical component of the HRQoL. Among them, five variables, namely the 6MWD (r = 0.44), SRI-MF (r = −0.40), fear of falling (r = −0.37), ST from LSP (r = −0.35), and handgrip strength (r = 0.30) exhibited relatively higher correlations with the physical component, and variables such as physical performances [5,16,17,18], mental-related health [6], and falling risk [11,13] are well-known to deteriorate the HRQoL. Therefore, to evaluate the physical component of the HRQoL in elderly adults, we recommend considering variables that can evaluate the functional endurance, muscle strength, stress levels, and falling risk; these variables include 6MWD, ST from LSP, handgrip, and SRI-MF. Thus, the study findings can provide reliable and objective results to enhance the understanding of the physical component of the HRQoL in elderly adults, and the intervention programs need to consider these factors to improve the physical component of the HRQoL.

### 4.3. HRQoL in Elderly Adults Based on the Mental Component

In the case of the mental component, five variables were used to form the best subset of the features. The demographic characteristics and questionnaire variables comprised of five features, namely the total number of diseases, SRI-MF score, total ISI score, and fear of falling, which are similar with previously reported results. The SF-36 includes several subscales of related mental evaluation, such as vitality, social functioning, role limitations caused by emotional problems, and mental health [9,35]. Additionally, the SRI-MF evaluates stress levels considering somatization, anger, and depression [40]. Thus, the low level of mental health indicates worse stress levels in elderly adults. Our results demonstrated that the relationship between the mental component and SRI-MF exhibited the highest correlation value (r = −0.51) in comparison with other variables. Furthermore, elderly adults with insomnia may be affected by mental disorders, such as depression and anxiety [75]. Similar results have been reported previously, wherein the ISI score was associated with the SRI-MF score, indicating that severe insomnia may worsen stress levels [29]. The risk of diseases also affects mental health, which can reduce the HRQoL in terms of both physical and mental components [6,8].

Furthermore, the 5 times sit-to-stand exercise assesses the lower body strength, which indicates a better performance as the completed time is faster. According to a previous study, the mental component may be associated with sarcopenia owing to self-perceived anxiety and depression. This is because individuals with sarcopenia may exhibit reduced mobility functions caused by the loss of strength and muscle mass, which can increase the fear of falling [76]. As sarcopenia-related index was not considered in this study, the decline in mental health can be associated with the decreased physical performance functions. Additionally, fear of falling affects in lowering the HRQoL considering the mental components in elderly adults [12,13]. Our study also indicates that the fear of falling is an important variable for predicting HRQoL considering the mental component in elderly adults, which is similar with previous studies [59,60,61]. Therefore, to evaluate the mental component of the HRQoL in elderly adults, we recommend considering additional variables apart from the mental-health-related variables. These variables need to evaluate functions, such as stress, insomnia severity, number of diseases, lower body strength, and fear of falling, which can be used to understand the HRQoL in terms of the mental component and can thereby serve as a reference.

### 4.4. Limitations and Future Applications

Our study demonstrated several strengths of the HRQoL in elderly. First, we evaluated the HRQoL in 775 elderly adults considering both the physical and mental components based on 96 independent variables, such as demographic characteristics, gait ability, and physical fitness, using the ML approach. We determined the best features among the 98 variables in terms of both the physical and mental components. This multidisciplinary approach could provide essential information on the importance of variables to enhance the understanding of the HRQoL in elderly. Furthermore, the study findings can be utilized by intervention programs as reference values to improve the physical and mental health of elderly adults. The intervention programs to improve specific factors (e.g., muscle strength, stress level, gait ability, and etc.) can help to effectively enhance theirs daily QoL in general. However, we also recognized several limitations of this study that need to be addressed. We predicted the HRQoL using the ML approach with only 775 samples, which may be relatively insufficient to normalize the model. Additionally, we did not consider the essential HRQoL model based on disease characteristics, such as cardiovascular diseases, neurodegenerative diseases, and musculoskeletal diseases. Therefore, we believe that a higher number of samples than that used in our study can enhance the reliability of the model and establish an accurate ML model considering different types of disease characteristics. Finally, our study determined that the ST from LSP is a significant independent variable that can evaluate the HRQoL in terms of the physical component in elderly adults. Although this variable can evaluate the lower body strength in elderly adults, sitting on the floor is uncommon in most countries, except East Asian countries. Therefore, if the ST from LSP is a necessary task to evaluate the HRQoL considering the physical component, it should be validated for various races and cultures, such as European, North American, South American, African, and Asian, to accurately evaluate the lower body strength in elderly adults.

## 5. Conclusions

Our study determined the best prediction models to identify the HRQoL in elderly adults in terms of both the physical and mental components based on the demographic characteristics, questionnaires, gait ability, and physical fitness using the ML approach. Feature selection worked well for the both components. This contributed to increase the performance and reduce the complexity of the model. Consequently, the approach of the ML algorithm for analyzing the HRQoL dataset is effective. In the case of the physical component, 11 variables were used to form the best subset of the features. We recommend performing several tasks to evaluate the functional endurance, muscle strength, stress level, and falling risk to effectively estimate the physical component of the HRQoL in elderly adults. Furthermore, in the case of the mental component, five variables were used to form the best subset of the features. Therefore, both mental-health-related and additional variables must be used to evaluate functions, such as stress, insomnia severity, number of diseases, lower body strength, and fear of falling for determining the mental component of the HRQoL. These findings can provide more reliable and objective results and improve the understanding of HRQoL in terms of both the physical and mental components in elderly adults, and we recommend considering these factors to improve their HRQoL in elderly undergoing intervention programs.

## Figures and Tables

**Table 1:** Statistics of the generated models which minimizes the average root mean square error (aRMSE) in both physical component and mental component.

Component	Model	CNoF	aRMSE	Std RMSE	Min RMSE	RMSE 25%	RMSE 50%	RMSE 75%	Max RMSE
Physical	LR_PV	33	13.92776	0.68349	11.96545	13.50805	13.95991	14.44462	15.38411
	(a) LR_FI	15	13.76088	0.71617	11.86612	13.29279	13.78654	14.24163	15.43223
	RF_PV	31	14.39872	0.73950	12.57450	13.91250	14.33669	14.98490	15.94272
	RF_FI	15	14.20864	0.68617	12.62523	13.68770	14.26701	14.74725	15.90077
	SVM_PV	8	14.76165	0.81308	12.99783	14.15996	14.79660	15.25064	17.17015
	SVM_FI	8	14.39575	0.82367	12.58128	13.84777	14.39586	15.01643	16.60752
Mental	(b) LR_PV	12	11.36805	0.61973	9.53575	10.89313	11.37711	11.78536	12.96427
	LR_FI	6	11.37025	0.70205	9.17258	10.86453	11.36144	11.82792	13.24375
	RF_PV	18	11.64159	0.59699	10.09463	11.21322	11.66051	12.00546	13.00478
	RF_FI	39	11.60706	0.62697	10.11383	11.11899	11.63986	11.98359	13.47048
	SVM_PV	4	11.79612	0.76560	10.14775	11.30421	11.70546	12.23328	13.82748
	SVM_FI	2	11.68649	0.75505	10.05444	11.15436	11.58823	12.22781	13.59275

In the case of the physical component, model (a) with cumulative number of features (CNoF) of 15 shows the best performance among all the models that can be generated. In the case of the mental component, model (b) with CNoF of 12 minimizes the aRMSE among all the models that can be generated.

**Table 2:** Description of beta of features in the best performing model.

Physical Component (LR_FI, CNoF = 15).	Beta	Mental Component (LR_PV, CNoF = 12)	Beta
Demographic characteristics		Demographic characteristics	
Age *	−1.88	Total number of diseases *	−2.78
Total number of diseases *	−3.63		
Questionnaires		Questionnaires	
Stress response index-modified form (SRI-MF) *	−6.98	SRI-MF *	−6.91
Total Insomnia severity index (ISI) *	−5.08	Total ISI *	−3.95
Total Physical activities (PAs) *	4.69	Fear of falling *	−3.71
Fear of falling *	−6.42		
Gait ability		Gait ability	
Preferred speed_coefficient of variance (CV) Single-support phase	−3.28	Faster speed_Walking speed	2.92
Faster speed_CV Single-support phase *	−3.53		
Slower speed_Stride time *	−0.54		
Physical fitness		Physical fitness	
6-min walking test (6MWT) *	7.66	6MWT	3.96
Standing time (ST) from a long sitting position (LSP) *	−6.02	Five times sit-to-stand *	−3.86
3-m Timed-up-and-go test right side	−6.83	3-m Timed-up-and-go test left side	−3.81
Single-leg stance	2.30	3-m Timed-up-and-go test right side	−3.61
Five times sit-to-stand	−6.06	Bicep curls right	3.33
Handgrip right side *	5.11	Bicep curls left	3.24
		ST from LSP	−2.83

The best performing models are the multiple linear regression (LR) model with the feature rank based on the feature importance (FI) with cumulative number of features (CNoF) of 15 for the physical component and the LR model with a feature rank based on the *p*-value (PV) with CNoF of 12 for the mental component. Features masked with a symbol, *, indicate that those features are selected for the best prediction model.

## Data Availability

The datasets generated and/or analyzed during the current study are not publicly available due to intellectual property reasons, but these are available upon a reasonable request.

## Supplementary Materials

The following are available online at https://www.mdpi.com/article/10.3390/ijerph182211816/s1, Table S1: Physical fitness test protocol; Table S2: Results of the feature rank; Table S3: Raw data; File S1: Detailed machine learning procedure.
