# Supplementary material for: Estimating Health-Related Quality of Life Based on Demographic Characteristics, Questionnaires, Gait Ability, and Physical Fitness in Korean Elderly Adults
Source: Int J Environ Res Public Health. 2021 Nov 11;18(22):11816. doi: 10.3390/ijerph182211816 (PMC8624167; doi:10.3390/ijerph182211816)
Supplement: Supplementary file 1 [file ijerph-18-11816-s001.zip › File S1. Detailed machine learning procedure..pdf]

International Journal of
Environmental Research and Public Health

MDPI

1. Methods

1.1. P-value calculation with One-way ANOVA

In a normal distribution with meanμ_j, and variance σ^2 with sample sizen_j, the appropriate model for the observations is One-way ANOVA which is$X_{ij} = \mu_j + e_{ij}$; i = 1, … , n_j, j = 1, … , b, i.e, for each j, X_{1j},… ,$X_{n_j j}$ are r.s. from N(μ_j, σ^2). P-value of each categorical feature is calculated by p-value = P(X≥ f-value), where f-value is calculated by following equation:

$$\text{f-value} = \frac{SS_{Between}/df_1}{SS_{Within}/df_2} = \frac{\sum_{j=1}^{b}\sum_{i=1}^{n_j}(\bar{X}_j-\bar{X})^2/b-1}{\sum_{j=1}^{b}\sum_{i=1}^{n_j}(X_{ij}-\bar{X}_j)^2/(\sum_{j=1}^{b}n_j)-b} \sim \mathrm{F}(df_1, df_2). \quad (1)$$

1.2. P-value calculation with Simple Linear Regression Model

With sample size n (i = 1,2,…,n), ε_i which is independent and identical distribution such that E(ε_i)=0 and Var(ε_i) = σ^2,Y_i is independent with E(Y_i) = $Y_i = \beta_0 + \beta_1 X_i$, and Var($Y_i$) = σ^2, where β_0, β_1 is regression coefficients to be estimated, simple linear regression is$Y_i = \beta_0 + \beta_1 X_i + \varepsilon_i$, i = 1,2, …, n. P-value of each continuous feature is calculated by p-value = P(X≥ f-value), where f-value is calculated by following equation:

$$\text{f-value} = \frac{SSR/df_1}{SSE/df_2} = \frac{\sum_{i=1}^{n}(\hat{Y}_i-\bar{Y})^2/1}{\sum_{i=1}^{n}(Y_i-\hat{Y}_i)^2/n-2} \sim \mathrm{F}(df_1 = 1, df_2 = n-2). \quad (2)$$

1.3. Gini Importance

The feature importance score provides the relative ranking of the spectral features and is a side product of random forest training. Gini importance refers to a probability that a particular feature will be selected for segmentation and a scale of the identification value for classification. This is Gini importance of an equation:

$$I_G(x) = \sum_T \sum_t \Delta i_x(t, T). \quad (3)$$

At each node t within the binary tree T of the random forest, the optimal split is found by using the Gini impurity i(t), which measures how well the potential split separates the two classes of samples at the particular node. The n_k samples from class k=[0,1] out of total of n samples at node t, the Gini impurity i(t) isi(t) = 1 – $(\frac{n_1}{n})^2$ – $(\frac{n_0}{n})^2$. The reduction Δi, which is the result of splitting and transmitting the sample into two sub-nodes t_a and t_b by a threshold t_x for the feature x, isΔi(t) = i(t) – $\frac{n_a}{n}$i(t_a) – $\frac{n_b}{n}$i(t_b).And the reduction of Gini impurity due to this optimal split Δi_x(t,T) is recorded and accumulated for all nodes t of all trees T in the random forest, individually for all features x.

1.4. False Discovery Rate

In the multiple comparison of the listed p-value, to control type I error, we reorganize it using the False Discovery Rate [S1]adjusting.*E(V/R)* ≤ *q*, where *q*, *0<q<1*, is a given constant.Let$p_1 \le p_2 \le \cdots \le p_k$be the p-values of *k* hypotheses, and *q* is given, and *ifj* = $max_{1\le i\le k}\{i \mid p_i \le \frac{i}{k}q\}$, then, FDR rejects hypotheses corresponding to$p_1 \le p_2 \le \cdots \le p_j$.

1.5. Multiple Linear Regression Model

Regression analysis is one of the most commonly used statistical method in dealing with the linear prediction [Yan, X, et al., 2009]. Multiple linear regression model explains the target as a linear combination of the features and weights. It can be used to quantify the relationship between the features and target. Population multiple linear regression

model relates a target(Y) to p -1 features($\mathbf{X}_1, \mathbf{X}_2, \ldots, \mathbf{X}_{p-1}$) is written as$\boldsymbol{Y}_i = \beta_0 + \beta_1 \boldsymbol{X}_{i1} + \beta_2 \boldsymbol{X}_{i2} + \cdots + \beta_{p-1} \boldsymbol{X}_{i,p-1} + \boldsymbol{\varepsilon}_i$, i = 1,2, … , n,by assuming that the ε_i which is an independent and identical distribution s.t. E(ε_i)=0, Var(ε_i) $= \sigma^2$,$\widehat{\boldsymbol{Y}} = \beta_0 + \beta_1 \boldsymbol{X}_1 + \beta_2 \boldsymbol{X}_2 + \cdots + \beta_{p-1} \boldsymbol{X}_{p-1}$.

1.6. Random Forest Regression

Random forest is the method based on bagging [Breiman, L., 1996] that aggregating trees trained by slightly different training data extracted from dataset under allowance of duplication. It is a technique that reduces the variance of an estimated prediction function. The concept is to improve the variance by curtailing the correlation between models without increasing the variance too much. Thus, it is especially suitable for high variance, low bias procedures such as trees [Dietterich, T. G.,2002]. It is achieved during the tree growth process through a random selection of input features. For regression, we fit the same regression tree multiple times to the bootstrap-sampled version of the training data and average the results [Liaw, A. et al, 2002]. The one of the biggest benefits of random forest is its generalization of performance based on decorrelation of trees [Breiman, L., 1996].

1.7. Support Vector Machine Regression of Radial Basis Function Kernel

Support Vector Machine [VAPNIK, Vladimir, et al., 1997] is one of the most adequate and widely used machine-learning tools, and has a competitive advantage over methods. It is based on the convex optimization problem, so that it does not cause a local minima problem. By function of kernel trick, it has been very successful in nonlinear classification, which is instead of computing the inner product in the high dimensionality of $\mathcal{H}$, which computes them using a radial basis function kernel [SCHOLKOPF, Bernhard, et al., 1997], K($\boldsymbol{x}_i, \boldsymbol{x}_j$) = {$\Phi(\boldsymbol{x}_i), \Phi(\boldsymbol{x}_j)$} = exp(-$\gamma \parallel \boldsymbol{x}_i - \boldsymbol{x}_j \parallel^2$), where Φ:$\mathbb{R}^p \rightarrow \mathcal{H}$, a function K: $\mathbb{R}^p \times \mathbb{R}^p \rightarrow \mathbb{R}$ such that for all x, y $\in \mathbb{R}^p$ in input space, and it helps speed up the computation. Then, it computes a linear support vector machine, but where the computation is carried out in some other place.

References

1. Benjamini, Y., & Hochberg, Y. (1995). Controlling the false discovery rate: a practical and powerful approach to multiple testing.Journal of the Royal statistical society: series B (Methodological),57(1), 289-300.
2. Yan, X., & Su, X. (2009).*Linear regression analysis: theory and computing*. World Scientific.
3. Breiman, L. (1996). Bagging predictors.Machine learning,24(2), 123-140.
4. Das, S. (2001, June). Filters, wrappers and a boosting-based hybrid for feature selection. In*Icml*(Vol. 1, pp. 74-81).
5. Dietterich, T. G. (2002). Ensemble learning.The handbook of brain theory and neural networks,2(1), 110-125.
6. Liaw, A., & Wiener, M. (2002). Classification and regression by randomForest.*R news*,*2*(3), 18-22.
7. Scholkopf, B., Sung, K. K., Burges, C. J., Girosi, F., Niyogi, P., Poggio, T., & Vapnik, V. (1997). Comparing support vector machines with Gaussian kernels to radial basis function classifiers.IEEE transactions on Signal Processing,45(11), 2758-2765.
8. Vapnik, V., Golowich, S. E., & Smola, A. (1997). Support vector method for function approximation, regression estimation, and signal processing.Advances in neural information processing systems, 281-287.
