# Supplementary material for: Estimating Health-Related Quality of Life Based on Demographic Characteristics, Questionnaires, Gait Ability, and Physical Fitness in Korean Elderly Adults
Source: Int J Environ Res Public Health. 2021 Nov 11;18(22):11816. doi: 10.3390/ijerph182211816 (PMC8624167; doi:10.3390/ijerph182211816)
Supplement: Supplementary file 1 [file ijerph-18-11816-s001.zip › IRB letter.pdf]

결과통지서

2019년 12월 23일에 접수된 연구종료보고서에 대하여 동아대학교 생명윤리위원회에서 심의하여 다음과 같이 결정하였음을 통지합니다.

**과제번호**	2-1040709-AB-N-01-201808-HR-023-08			**관리번호**	HR-023-08	
**연구과제명**	스포츠 평가기반의 맞춤형 시니어 피트니스 서비스 기술 개발					
**연구책임자**	성명	염 창 홍	소속	건강관리학과	직위	부교수

**심의대상**	□연구계획변경 □지속심의/중간보고 □중대한 이상반응<br>□위반/이탈사례 ■**연구종료**/결과보고 □기타( )
**심의일자**	2019년 12월 26일
**심의의견**	**- 연구과제의 종료를 승인합니다.**
**심의된 서류**	1. 연구종료보고서<br>2. 취득한 동의서 사본 6부

2020년 1월 2일

동아대학교 생명윤리위원장

동아대학교 생명윤리위원회위원장인

본 통지서에 기재된 사항은 동아대학교 생명윤리위원회에 기록된 내용과 일치함을 증명합니다.
본 위원회는 생명윤리 및 안전에 관한 법률과 관련 법규를 준수합니다.
본 연구와 이해상충(Conflict of Interest)이 있는 위원이 있을 경우 연구의 심의에서 배제하였습니다.
본 통지서의 사본은 동아대학교 생명윤리위원회에서 보관합니다.
