# Supplementary material for: Estimating Health-Related Quality of Life Based on Demographic Characteristics, Questionnaires, Gait Ability, and Physical Fitness in Korean Elderly Adults
Source: Int J Environ Res Public Health. 2021 Nov 11;18(22):11816. doi: 10.3390/ijerph182211816 (PMC8624167; doi:10.3390/ijerph182211816)
Supplement: Supplementary file 1 [file ijerph-18-11816-s001.zip › Table S1 Physical fitness test protocols.pdf]

Table S1. Physical fitness test protocols

Upper body strength

	Purpose	Equipment	Protocol
Grip strength	To assess upper body strength	Isometric digital handgrip dynamometer (T.K.K. 5401 Grip-D, Takei Scientific Instruments, Tokyo, Japan)	1. Participants stand with their shoulder adducted and neutrally rotated. Elbow is toward the body and flexed at 90 degrees and the forearm and wrist in a neutral position.
			2. The handle of the dynamometer is adjusted if required - the base should rest on the first metacarpal (heel of palm), while the handle should rest on the middle of the four fingers.
			3. When ready the participants squeeze the dynamometer with maximum isometric effort, which is maintained for about 5 seconds. No other body movement is allowed.
			4. The participants should be strongly encouraged to give maximum effort.
Biceps curls	To assess upper body strength	Dumbbell (3 kg for men; 2 kg for women)	1. Investigator asks participants to perform for 30 seconds, using the stopwatch.
			2. Do as many curls as participants can in the allotted 30-second time period, moving in a controlled manner.
			3. Do a full curl, squeezing lower arm against upper arm at the top of each curl, and returning to a straight arm each time. Keep upper arm still, and do not swing the weight.
			4. If participants have started raising the weight again and are over halfway up when time is called, it may count.
			5. Record the score on the scorecard.

Lower body strength

	Purpose	Equipment	Protocol
Five times sit-to-stand	To assess lower body strength	Chair without arms, Stopwatch	1. Place the chair against a wall where it should be stable.
			2. Sit in the middle of the chair with feet flat on the floor, shoulder-width apart, back straight.
			3. Cross arms at the wrist and place them against the chest.
			4. Do five times rise to a full stand and sit motion as soon as possible
Standing time from a long sitting position	To assess lower body strength	Stopwatch	1. Participants sit on the floor with knees extended.
			2. Participants stand up as quickly as possible to a stable erect position from a long sitting position on the floor.
			3. Record the time on the scorecard.

Lower body flexibility

	Purpose	Equipment	Protocol
Chair sit and reach	To assess lower body flexibility	Chair, Ruler	1. Place the chair against a wall where it should be stable.
			2. Slide forward in the chair until participants are able to straighten one of their legs. The ankle of the straight leg should be flexed at about a 90-degree angle. The other foot should be flat on the floor.
			3. Place one of the hands directly on top of the other so that participants are stacked with their fingers extended.
			4. Exhale as subject bend forward at the hip and try to reach their toes. If the extended leg begins to bend, move back in the chair until the leg is straight.
			5. Hold the stretch for at least 2 seconds and do not bounce or jerk as reach.
			6. Take reaches on each leg, determining which side is more - flexible. Recording only the most flexible side. The center of the toe of the shoe is considered to be a measurement of "0".

Static balance

	Purpose	Equipment	Protocol
Single-leg balance	To assess static balance	Stopwatch	1. Performed with eyes open and arms on the hips.
			2. Participants must stand unassisted on one leg and is timed in seconds from the time one foot is flexed off the floor to the time when it touches the ground or the standing leg, or an arm leaves the hips.
			3. Record the time on the scorecard.

Dynamic balance

	Purpose	Equipment	Protocol
3 m timed up and go	To assess dynamic balance	Chair, Cone, Stopwatch	1. Sit in the chair with hands-on thighs, feet flat on the floor with one foot slightly ahead of the other.
			2. Investigator signals, "go" and start the watch. For test accuracy, the investigator must start the watch on the signal, "go." Do not wait to start the watch after participants have started to move.
			3. The test is timed to the nearest tenth (0.1) of a second, so it is important to be as accurate as possible when starting and stopping the watch.
			4. Upon the signal "go" rise from the chair and walk as quickly as possible out to the marker. Participants may press off their thighs of the chair when they rise. Do not run. Walk around the outside of the marker and return to their seat as quickly as possible, being sure to be safe in their movements.
			5. As soon as they are fully seated again, the investigator should stop the watch and record their time to the nearest tenth of a second.

Functional (or cardiorespiratory) endurance

	Purpose	Equipment	Protocol
6 min walk distance	To assess functional (or cardiorespiratory) endurance	Chair, Cone, Stopwatch, Measurement scale	1. Place cones at either end of the 30 m stretches as turning points.
			2. The participants are to walk as far as possible for 6 minutes. They walk back in the hallway.
			3. Six minutes is a long time to walk, so they are exerting themselves. Participants are permitted to slow down, to stop, and to rest as necessary. They may lean against the wall while resting, but resume walking as soon as they are able.
			4. Participants should pivot briskly around the cones and continue back the other way without hesitation.
			5. Measure the best distance walked in the meter when timed out.
